# Men and Their Children

**Published:** 1881-02

**Authors:** 


### MEN AND THEIR CHILDREN.

Just at an age when a man begins to get himself well in hand, to grow broader in his view, sweeter in his temper, to lose the acridity, the positiveness, the inability of youth to generalize the detailed experience he has gained—to be fit, in a word, to accomplish the work he had planned to do in the world—he begins, if he is a father, to set himself wholly on one side for the sake of the future little men and women about his table. His great picture is never painted, his epic is never written, the best work of which he is capable is never done; he gives himself up to pot-boilers in order to bring up another man, who perhaps may be inferior to himself. This is the work which has been going on since the beginning of the world. We make much of the pelican, who robs her breast of a few drops of blood for her young, but the great rule of humanity has been that one generation of middle-aged people sacrificed their chances, their hopes, their work for the world, for their children. The great oak crumbles and dies that the ground may be richer for the sapling. It is a just sequence. But it may be carried too far, and it is carried farther in America than in any other country. Fathers and mothers have a right of development which they themselves are bound to respect. A man will be the better able to elevate his children if he stops his daily suicidal grind long enough to consider that he also is a human being, whose character and work in the world will probably be quite as helpful as the boy's for whom he is sacrificing all his time and opportunities. One is sometimes tempted to wonder whether in the lives to come there will not be some place where the ambitions and hopes and thwarted possibilities of the middle-aged may have the chances which here, fitly enough, are reserved for the young.—*New York Tribune.*

---

Europe is still interested in the Italian government's latest method of raising money. For the future a person will pay $6,000 on being created an Italian prince, and $5,000 on being made a duke. The charge for a marquisate is $4,000; for the title of "conte," $3,000; of baron or viscount, $2,000. Any other noble title will cost $1,000. Should the person enobled desire that the title shall not descend to any successor, a discount of two-fifths on the regular tariff will be allowed. For a grant of arms, etc., $140 will be charged.

